# Stepwise Sous-Vide Cooking as a Novel Approach to Enhance the Water-Holding Capacity and Tenderness of Chicken Breast

**DOI:** 10.3390/foods14101708

**Published:** 2025-05-12

**Authors:** Sin-Woo Noh, Dong-Heon Song, Hyun-Wook Kim

**Affiliations:** 1 Division of Animal Bioscience & Integrated Biotechnology, Gyeongsang National University, Jinju 52828, Republic of Korea; nohsinwoo@naver.com (S.-W.N.); timesoul@naver.com (D.-H.S.); 2 Department of GreenBio Science, Gyeongsang National University, Jinju 52725, Republic of Korea

**Keywords:** heating rate, shear force, stepwise cooking, tenderness, water-holding capacity

## Abstract

Optimal sous-vide and multiphase cooking strategies remain underexplored despite their potential to improve the tenderness and juiciness of chicken breast. This study aimed to optimize sous-vide cooking conditions (Experiment I) and evaluate the effects of stepwise sous-vide cooking on the quality attributes (Experiment II). In Experiment I, a two-factor, three-level central composite design was employed to optimize the temperature (50, 60, and 70 °C) and time (3, 4.5, and 6 h) using response surface methodology. The optimal condition (55 °C for 3 h) significantly reduced cooking loss (11.47%) and shear force (11.84 N). In Experiment II, five cooking conditions were compared: conventional control (75 °C/30 min), sous-vide control (55 °C/180 min), and three stepwise methods (Stepwise I, 45 °C/180 min + 55 °C/180 min; Stepwise II, 55 °C/180 min + 75 °C/8.5 min; and Stepwise III, 55 °C/180 min + 95 °C/3 min). Stepwise II and III increased cooking loss (16.4% and 20.5%, respectively) and reduced moisture (*p* < 0.05), but Stepwise II significantly decreased shear force (12.50 N), retaining moisture comparable to conventional control (17.35 N). Stepwise sous-vide cooking, particularly Stepwise II, appears promising for enhancing tenderness without causing excessive water loss. Further research should evaluate the sensory properties and microbiological safety for potential practical applications.

## 1. Introduction

Sous-vide cooking, which involves precise temperature control at relatively low temperatures in vacuum-sealed packaging for a long time, is well-known to improve the eating quality of tough meats [1]. This technique is also advantageous for lean meats such as chicken breast, which inherently contains low intramuscular fat and is prone to a less juicy and tough texture when improperly cooked [2]. Although the positive effects of sous-vide cooking, especially improvements in tenderness and water-holding capacity, are well recognized, considerable variability exists in the literature regarding optimal processing conditions for chicken breast, particularly in terms of temperature–time combinations: 60 °C for 2–3 h [2], 60 °C for 1 h [3], 60 °C for 2 h [4], and 80 °C for 2 h [5]. This inconsistency implies the need for further research to optimize the sous-vide parameters for achieving desirable quality attributes in chicken breast.

The thermal denaturation of muscle proteins during heating is a critical factor influencing the desirable texture and mouthfeel of cooked meat. The denaturation temperatures of major muscle proteins in chicken breast are well documented: myosin at 62 °C, collagen at 70 °C, and actin at 82 °C [6]. Sous-vide cooking, typically conducted at around 60 °C, minimizes excessive protein denaturation and shrinkage while promoting the gelatinization of connective tissues, resulting in the enhancement of tenderness and water-holding capacity [2]. Recent findings by Noh et al. [7], who investigated optimal sous-vide cooking conditions, demonstrated that increasing the cooking temperature from 55 °C to 65 °C led to improved tenderness in chicken breast, despite increased cooking loss. Thus, careful consideration is required when adjusting sous-vide temperatures to consistently improve meat tenderness, especially given the contradictory effects of temperature changes on tenderness and water-holding capacity.

In terms of the efficiency and consistency of sous-vide cooking, recent research has explored stepwise sous-vide cooking, indicating multi-phase heating protocols sequentially combining low and high temperatures, as a method of further improving both tenderness and water-holding capacity [8]. In tough beef and goat muscles, the scientific rationale for this approach involves the activation of the proteolytic activity of endogenous proteases at 40–50 °C, followed by continuous sous-vide cooking at higher temperatures (60–70 °C) [8,9]. For chicken breast, Noh et al. [7] recently proposed a novel stepwise concept that includes terminating the cooking at a conventional high temperature after the initial sous-vide cooking to further improve tenderness without compromising water-holding capacity. However, there is currently nothing in the literature comparing such stepwise sous-vide cooking methods to guarantee improvements in tenderness and water-holding capacity in chicken breast.

Considering the expanding industrial application of sous-vide cooking, particularly within the poultry sector, developing a modified sous-vide technique that ensures improved tenderness and water-holding capacity compared to single-step sous-vide cooking is crucial for achieving consistent quality enhancement in cooked meat products. Therefore, the objectives of this study were to establish the sous-vide cooking conditions (temperature and time) that enhance tenderness and water-holding capacity compared to conventional cooking (e.g., boiling) through response surface methodology, and to evaluate the effects of stepwise sous-vide cooking methods on the general meat quality attributes of chicken breast. These stepwise methods include an initial low-temperature treatment followed by sous-vide cooking, which is designed to activate the intrinsic protease activity, and an initial sous-vide treatment followed by high-temperature cooking, intended to induce collagen denaturation.

## 2. Materials and Methods

### 2.1. Experimental Design

This study was designed to evaluate the optimization of the sous-vide cooking parameters and to assess the effects of stepwise sous-vide cooking techniques on the quality of chicken breast through two separate experimental approaches. In Experiment I, a two-factor, three-level, face-centered central composite design was employed, with five replicates (six breasts/treatment/replicate) at the center point. The design, based on previous observations [2,7], investigated the effects of temperature (*X*_1_, 50–70 °C) and time (*X*_2_, 3–6 h) on two response variables (cooking loss and shear force) across 13 experimental runs (Table 1). The three coded levels (−1, 0, +1) for both temperature and time were used to set up these 13 runs. The effects of these factors (*X*_1_ and *X*_2_) on the two response variables (*Y*) were modeled using second-order polynomial regression equations: *Y* = *β* + *β*_1_*X*_1_ + *β*_2_*X*_2_ + *β*_3_*X*_1_*X*_2_ + *β*_4_*X*_1_^2^ + *β*_5_*X*_2_^2^, where *β*_0_ is a constant, *β*_1_ and *β*_2_ are the coefficients for the linear effects of the independent variables, *β*_3_ is the coefficient for the interaction between the variables, and *β*_4_ and *β*_5_ are the coefficients for the quadratic effects of the independent variables. To validate the optimized condition, additional experimental runs with three independent replicates (six breasts/replicate) were conducted.

In Experiment II, a completely randomized block design with a total of three independent batches was used to determine the effects of stepwise sous-vide cooking methods on chicken breasts. In each batch, thirty chicken breasts (*M. pectoralis major*) were obtained from fifteen chicken broiler carcasses, which were randomly assigned to five different cooking treatments (six breasts/treatment/batch). A total of three independent batches (blocks) were performed.

### 2.2. Sous-Vide Cooking Procedure

Fresh chicken broilers were purchased from a local market, and the chicken breasts were manually deboned and weighed. The average pH of all left-side chicken breast samples before cooking, which had an average weight of 125.42 ± 6.28 g, was 5.95 ± 0.15, with no significant differences among the treatment groups. The chicken breast samples were individually placed in vacuum-packaging pouches (nylon/polyethylene, 150 × 200 mm, 65 μm thickness), vacuum-sealed at −100 kPa, and placed in a 4 °C refrigerator to equilibrate to the same initial temperature for 6 h. In Experiment I, the vacuum-sealed samples were cooked at the targeted temperature and time conditions (Table 1) using a precision-controlled digital water bath (JSIB-22T, JS Research Inc., Hwaseong, Republic of Korea). After cooking, the samples were cooled at room temperature for 1 h and then used for further analysis.

**Figure 1:**
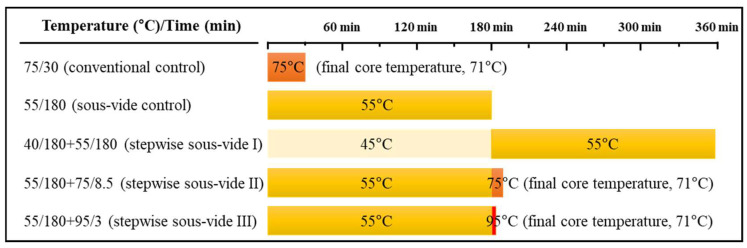
A schematic diagram showing the cooking conditions (time and temperature) of stepwise sous-vide cooked chicken breasts. The control was cooked at 75 °C for 30 min, and the sous-vide (SV) control was cooked at 55 °C for 3 h. The stepwise sous-vide treatments were cooked at 55 °C for 3 h before/after the combined cooking treatments at 40, 75, and 95 °C, respectively. The final core temperature of the control, stepwise SV II, and stepwise SV III was set equally to 71 °C.

In Experiment II, five different cooking conditions were compared to evaluate the efficacy of stepwise sous-vide cooking. A schematic diagram of the cooking conditions is shown in Figure 1. The conventional control samples, vacuum-packaged in the same manner above, were cooked in a water bath set at 75 °C for 30 min with the internal temperature monitored until it reached 71 °C. The internal temperature was measured using an insert-type digital thermometer (Tes-1305, Tes Electrical Corp., Taipei, Taiwan) equipped with an iron–constantan thermocouple. Meanwhile, the sous-vide control samples were cooked in a water bath set at 55 °C for 180 min, which was the optimal condition suggested through the result of Experiment I. Three stepwise sous-vide cooking treatments were conducted as follows: cooking in the water bath set at 40 °C followed by sous-vide cooking at 55 °C for 180 min (stepwise sous-vide I), sous-vide cooking at 55 °C for 180 min followed by cooking in a water bath set at 75 °C for 8.5 min (stepwise sous-vide II), and sous-vide cooking at 55 °C for 180 min followed by cooking in a water bath set at 95 °C for 3 min (stepwise sous-vide III). For the stepwise sous-vide II and III treatments, the final core temperature was set to 71 °C, as in the case of the conventional control above. The additional cooking times after sous-vide cooking required to reach the targeted internal temperature were determined through preliminary experiments, since it was not possible to monitor the internal temperature directly in vacuum-sealed samples. The cooking rates at 75 °C and 95 °C after the sous-vide cooking were calculated as 1.88 °C/min and 5.33 °C/min, respectively. In the case of stepwise sous-vide cooking, the samples were immediately transferred to water baths set at the designated temperatures for each step of the experiment, without additional treatment. All cooked samples were cooled at room temperature for 1 h and then used for further analysis. Each breast (biological replicate) had three technical replicates.

### 2.3. Analysis of Sous-Vide Cooked Chicken Breast

#### 2.3.1. Cooking Loss

The weight of the raw chicken breast was measured before packaging. After cooking and cooling, the cooked sample was removed from the packaging pouches, blotted with paper towels to remove surface moisture, and then re-weighed. The cooking loss was calculated as the percentage difference in weight between the raw and cooked samples using the following equation: Cooking loss (%) = ((weight of raw sample (g) − weight of cooked sample (g))/weight of raw sample (g) × 100.

#### 2.3.2. Shear Force

The shear force of the cooked samples was measured to assess the instrumental tenderness, according to the method of Song et al. [4]. Five rectangular strips (4.0 × 1.0 × 1.0 cm^3^) were manually cut from the superior side of the cooked chicken breast, each diagonally along the muscle fibers. The middle of each strip was severed using a Warner–Bratzler device (V-blade) on a texture analyzer (CT3, Brookfield Engineering Laboratories, INC., Middleboro, MA, USA). The test speed was set at 2 mm/s, and the maximum peak force (N) of the strips was averaged.

#### 2.3.3. Moisture Content

The moisture content of the cooked chicken breast was measured in triplicate, according to the oven air-drying method [10].

#### 2.3.4. pH Measurement

The pH of the sample was measured at three random spots using an insert-type pH meter (HI99163, Hanna Instruments, Woonsocket, RI, USA). The pH meter was calibrated with standard buffer solutions at pH 4.01 and 7.00 (Thermo Fisher Scientific, Waltham, MA, USA) before use.

#### 2.3.5. SURFACE Color Characteristic

The surface characteristics of the cooked chicken breasts were measured using a colorimeter (CR-400, Minolta, Osaka, Japan) equipped with an 8 mm diameter aperture, a 2° standard observer, and a D_65_ illumination source. The colorimeter was calibrated using a manufacturer’s white tile (CIE L*: +93.01, CIE a*: −0.25, CIE b*: +3.50). The cooked and cooled samples were exposed to the atmosphere for 1 h, and CIE L*, a*, and b* values were obtained from random spots on the skin side and used to calculate the hue angle and chroma [11].

$$Hue\;angle=arctangent(b^{*}/a^{*})$$

$$Chroma=\sqrt{a^{*2}+b^{*2}}$$

#### 2.3.6. Protein Solubility

The total protein solubility of the cooked samples was measured following the method of Warner et al. [12], as described by Noh et al. [7]. Two grams of the chopped sample were homogenized with 20 mL of 0.1 M potassium phosphate buffer (pH 7.2) containing 1.1 M potassium iodide at 12,000 rpm for 2 min (HG-15A, Daihan Sci., Seoul, Republic of Korea). The homogenate was stored in a refrigerator at 2 °C overnight, then centrifuged at 1500× *g* for 20 min at 4 °C and filtered through Whatman no. 1 filter paper. The protein concentration of the filtered supernatant was determined using the Biuret method, and the protein solubility was expressed as milligrams of soluble protein per gram of sample (mg/g).

#### 2.3.7. Total Collagen Content

The total collagen content of the samples was measured following the method described by Starkey et al. [13], with slight modifications described by Noh et al. [7]. One hundred milligrams of the chopped sample were mixed with 3 mL of 3.5 M sulfuric acid and hydrolyzed in a drying oven at 105 °C for 16 h. The hydrolysate was diluted to 50 mL with distilled water and then filtered through Whatman no. 1 filter paper. One milliliter of the filtrate was mixed with 3.75 mL of distilled water and 0.25 mL of 1.5 M NaOH, and 0.5 mL of this mixture was reacted with 0.25 mL of an oxidant solution (50 mM chloramine-T hydrate, 210 mM citric acid, 375 mM NaOH, 661 mM sodium acetate trihydrate, and 29% *v*/*v* 1-propanol, pH 6) for 20 min. After the reaction, the mixture was combined with 0.25 mL of coloring reagent (246 mM 4-dimethylaminobenzaldehyde, 35% *v*/*v* perchloric acid, and 65% *v*/*v* 2-propanol) and heated in a water bath at 60 °C for 15 min. After cooling for 3 min, the absorbance of the mixture was measured at 558 nm, and the total collagen content was determined using a standard curve prepared with hydroxyproline solutions of 0.6, 1.2, 1.8, and 2.4 μg/mL. The total collagen content was calculated using the following equation: Total collagen content (mg/g dry matter) = (hydroxyproline (μg/mL) × 7.25/1000)/(sample weight (mg)/250).

#### 2.3.8. Myofibrillar Fragmentation Index (MFI)

The MFI was measured using the method of Noh et al. [7]. Four grams of the sample were homogenized with 40 mL of isolation buffer (100 mM KCl, 20 mM K-phosphate, and pH 7.0, 1 mM NaN_3_) for 30 s, then centrifuged at 1000× *g* for 10 min. The residue was resuspended in 20 mL of isolation buffer. This process was repeated twice under the same conditions. The connective tissue was removed by filtering the suspension through an 18-mesh polyethylene filter, followed by additional centrifugation. The protein concentration was measured using the Biuret method after the centrifugation process was repeated twice more, each time suspending the filtered mixture in 20 mL of isolation buffer. The obtained myofibrillar protein was diluted to a concentration of 0.5 ± 0.05 mg/mL with isolation buffer. The absorbance was measured at 540 nm, and the MFI was calculated by multiplying the absorbance value by 200.

#### 2.3.9. Lipid Oxidation

Lipid oxidation was determined according to the 2-thiobarbituric acid reactive substance (TBARS) method described by Noh et al. [7]. Five grams of the chopped sample were mixed with 15 mL of distilled water and 100 µL of 6% 2,6-di-tert-butyl-4-methylphenol (BHT) and then homogenized at 12,000 rpm for 15 s. Two milliliters of the homogenate were mixed with 4 mL of TBA solution (20 mM 2-thiobarbituric acid in 15% trichloroacetic acid). The mixture was heated at 80 °C for 15 min in a water bath and then cooled in cold water for 10 min. After cooling, the sample was mixed using a vortexer and then centrifuged at 2000× *g* for 10 min at 25 °C (Combi- 514R, Hanil SME, Anyang, Republic of Korea). The supernatant was filtered through Whatman no. 4 filter paper, and the absorbance of the filtrate was measured at 531 nm using a spectrophotometer (Libra S22, Biochrome, Cambridge, UK). The TBARS value was calculated using the following equation: TBARS value (mg MDA/kg sample) = 5.3102 × Absorbance_531nm_ + 0.0356.

#### 2.3.10. Protein Oxidation

Protein oxidation was determined by evaluating the carbonyl formation, according to the colorimetric method using 2,4-dinitrophenylhydrazine (DNPH) described by Soyer et al. [14]. The carbonyl content was expressed as nmol carbonyls per mg protein (nmol/mg protein).

### 2.4. Statistical Analysis

In Experiment I, the experimental design, visualization, and optimization of the response surface methodology were performed using Minitab statistical software (version 17.0). In Experiment II, data on the quality attributes were statistically analyzed using the general linear model (GLM) procedure of SPSS 18.0 software (SPSS Inc., Chicago, IL, USA), in which treatment was fixed as the main effect. Duncan’s multiple range test was used to compare the mean values (*p* < 0.05).

## 3. Results and Discussion

### 3.1. Optimization of Sous-Vide Cooking Conditions (Experiment I)

**Figure 2:**
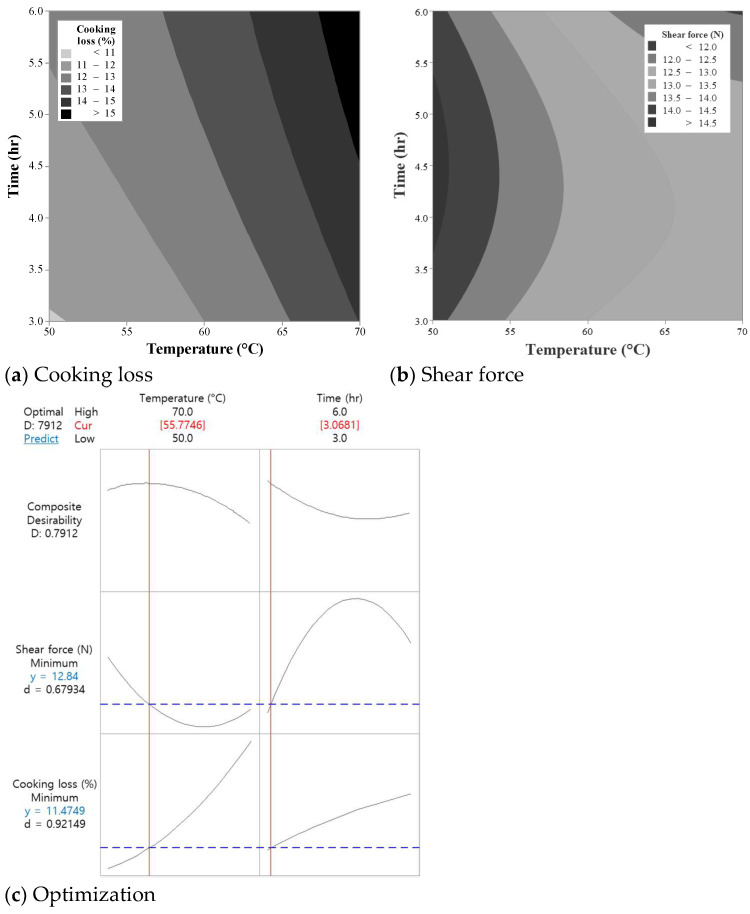
Contour plots presenting the effects of cooking temperature and time on cooking loss (**a**) and shear force (**b**), and optimization of sous-vide cooking conditions of chicken breasts (**c**). The range of optimal cooking conditions (temperature and time) was limited within 50–70 °C and 3–6 h, respectively. In the optimization graph (**c**), the X-axis represents the independent variable, and the Y-axis represents the response value, respectively.

Previous studies have shown considerable variation in optimal temperature–time combinations for improving tenderness and water-holding capacity of chicken breast, where experimental approaches of factorial arrangement and completely randomized block design were mostly considered to compare the effects of various temperature–time combinations on the meat quality attributes [2,3,7]. Response surface methodology (RSM) efficiently optimizes multiple food-processing variables and their interactions, making it a powerful tool for improving product quality and process efficiency. Thus, in Experiment I, the response surface methodology with the face-centered central composite design was used to optimize the temperature–time combinations in sous-vide cooking of chicken breast. As the critical factors affecting the quality attributes of sous-vide cooked chicken breast, cooking loss and shear force were investigated with a total of 13 experimental runs (Table 1) where the settings of temperature (50–70 °C) and time (3–6 h) was based on the results of previous studies [2,7]. At the experimental settings, the cooking loss and shear force of the sous-vide-cooked chicken breasts ranged from 11.13 to 15.48% and 11.81 to 14.94 N, respectively. The contour plots showing the effects of the temperature and time on the cooking loss and shear force of the chicken breasts are shown in Figure 2a,b. The variance of the regression coefficient calculated for optimizing the sous-vide cooking conditions of chicken breasts is presented in Table 2.

#### 3.1.1. Effects on Cooking Loss

The linear model for cooking loss was significant (*p* = 0.004), with the linear term for temperature (*X*_1_) contributing to the variation in cooking loss (Table 2 and Figure 2a). Temperature was found to have a more pronounced linear effect on cooking loss compared to time (*p* < 0.05). As the temperature increased from 50 °C to 70 °C, the cooking loss significantly increased (*p* < 0.05), likely due to increased protein denaturation and shrinkage. This is consistent with previous findings by Park et al. [2] and Noh et al. [7], who reported similar trends in cooking loss for sous-vide cooked chicken breasts. In contrast, time (*X*_2_) had a less-significant effect on cooking loss (*p* > 0.05), with a numerical increase observed as the cooking time extended from 3 to 6 h. No significant interaction between temperature and time (*X*_1_*X*_2_) was found. Similarly, Park et al. [2] found no interaction effects of temperature (60 and 70 °C) and time (1, 2, and 3 h) on the cooking loss of sous-vide cooked chicken breast.

#### 3.1.2. Effects on Shear Force

The shear force value, which is instrumentally indicative of meat tenderness, was significantly affected by both temperature and time (Table 2 and Figure 2b). Temperature exhibited a strong linear effect on shear force (*p* = 0.004). As the cooking temperature increased from 50 °C to 70 °C, the shear force decreased significantly, probably indicating improved tenderness. This trend can be attributed to the gradual solubilization of collagen molecules at higher temperatures, as previously observed by Noh et al. [7]. The quadratic effect of time (*X*_2_^2^) was also significant (*p* = 0.034). An increase in cooking time from 3 to 6 h resulted in a modest decrease in shear force (*p* < 0.05). In addition, the interaction term (*X*_1_*X*_2_) for shear force was statistically significant, indicating that the effect of time on tenderness was inconsistent depending on the temperature range.

#### 3.1.3. Optimization and Validation

The optimization plot of the temperature and time in sous-vide chicken breast is shown in Figure 2c. The RSM model predicted that the optimal sous-vide cooking conditions for minimizing cooking loss and shear force (maximizing tenderness) were achieved at a temperature of approximately 55 °C and a cooking time of 3 h. Under these conditions, the model predicted a cooking loss of 11.47% and a shear force value of 12.84 N, representing the best compromise between water-holding capacity and tenderness. To validate the RSM model, additional experimental runs were conducted at the predicted optimal conditions. The experimental values for cooking loss and shear force were 11.52% and 12.64 N, respectively, and no significant difference between the predicted and measured values was observed. The strong agreement between the predicted and observed values confirms the reliability of the RSM model in predicting the optimal sous-vide cooking conditions for chicken breasts.

### 3.2. Comparisons of Stepwise Sous-Vide Cooking Conditions (Experiment II)

#### 3.2.1. Effects on pH and Surface Color Characteristics

The pH and color of chicken breast subjected to stepwise sous-vide cooking are presented in Table 3. The pH of the sous-vide cooked samples, which ranged from 6.23 to 6.34, was not influenced by the stepwise sous-vide cooking treatments (*p* > 0.05). In terms of the color characteristics, sous-vide-cooked meat is typically lighter and redder than conventionally cooked meat [2] and often exhibits a pinkish color due to the limited thermal denaturation of myoglobin at lower cooking temperatures [7]. In this study, the sous-vide cooking method significantly affected the surface color of chicken breast. However, no significant differences in hue angle and chroma were found (Table 3). The sous-vide control exhibited lower CIE L* (lightness) values but higher CIE a* (redness) than the conventional control (*p* < 0.05). For stepwise SV I treatment, the color parameters remained consistent with those of sous-vide control (*p* > 0.05), indicating no substantial changes in color due to this stepwise approach. However, the additional heating process in the stepwise SV II and III treatments resulted in increased lightness and decreased redness, aligning the color characteristics of the treatments more closely with those of the conventional control (*p* > 0.05). The denaturation of intramuscular myoglobin occurs at 55 °C, which contributes to a reduction in redness and an increase in yellowness in cooked meat [15]. Thus, the observed results are likely attributable to further thermal denaturation of myoglobin in the stepwise SV II and III treatments, as the additional heating raised the core temperature to match that of the conventional control (71 °C). Our findings suggest that, while the initial phase of stepwise sous-vide cooking may maintain the characteristic redness associated with sous-vide methods, subsequent increases in temperature diminish this effect by promoting myoglobin denaturation.

#### 3.2.2. Effects on Water-Holding Capacity (Cooking Loss and Moisture Content)

**Figure 3:**
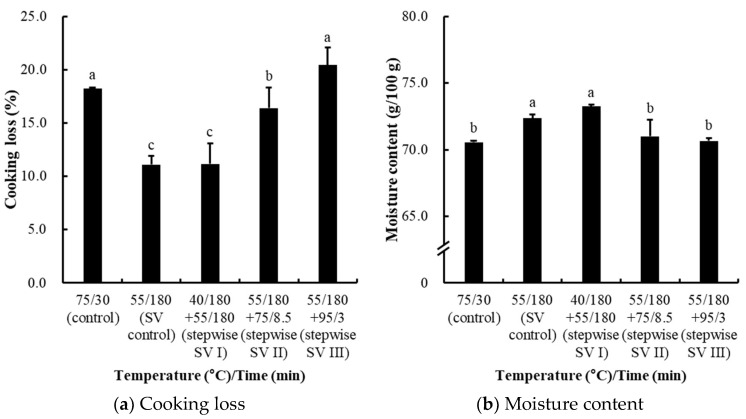
Cooking loss (**a**) and moisture content cooked sample (**b**) of stepwise sous-vide cooked chicken breasts. The control was cooked at 75 °C for 30 min, and the sous-vide (SV) control was cooked at 55 °C for 3 h. The stepwise sous-vide treatments were cooked at 55 °C for 3 h before/after the combined cooking treatments at 40, 75, and 95 °C, respectively. The final core temperature of the control, stepwise SV II, and stepwise SV III was set equally to 71 °C. Error bar means standard deviation. a–c Means sharing the same letters between treatments are not significantly different (*p* ≥ 0.05).

The cooking loss and moisture content of chicken breasts subjected to stepwise sous-vide cooking are presented in Figure 3. The lowest cooking loss was observed in the sous-vide control (11.10%) and the stepwise SV I treatment (11.16%) (*p* < 0.05; Figure 3a). When compared to the conventional control, this indicates that these temperature and time combinations could be effective in minimizing water loss during cooking. Conversely, the additional heating after sous-vide cooking in the stepwise SV II and III treatments exhibited greater cooking loss than the sous-vide control. However, for the stepwise SV II treatment, there was a noticeable reduction in cooking loss compared to the conventional control (*p* < 0.05), suggesting that the heating rate after sous-vide cooking might still be a crucial factor influencing water migration in cooked chicken breast. Moisture content was highest in the sous-vide control and stepwise SV I treatment (*p* < 0.05; Figure 3b), showing a well-known strong correlation with the reduced cooking loss. In the stepwise II and III treatments, moreover, the decreased moisture content was also observed (*p* < 0.05).

Myofibrillar protein undergoes thermal denaturation during cooking, where lateral shrinkage occurs at temperatures between 40 °C and 60 °C, expanding the gap between myofibrils and improving water retention. However, at temperatures between 60 °C and 65 °C, longitudinal shrinkage begins, leading to water exudation and loss [16,17,18]. Thus, the moisture retention observed at lower temperatures is primarily due to lateral expansion, while higher temperatures induce longitudinal shrinkage and subsequent moisture loss. Both cooking temperature and time influenced cooking loss, but cooking temperature had a more pronounced effect on myofibrillar protein shrinkage [19]. Park et al. [2] reported that sous-vide cooked chicken breast at 70 °C resulted in significantly higher cooking loss compared to 60 °C, and extended cooking times at the same temperature further increased cooking loss. This is consistent with our findings, where stepwise SV II and III treatments showed significantly higher cooking loss and decreased moisture content. These effects are likely due to the longitudinal contraction of proteins at elevated temperatures, which promotes water exudation. Our results also suggest that controlling the final temperature and heating rate could be essential for designing modified sous-vide cooking methods to improve the water-holding capacity in sous-vide-cooked chicken breast.

#### 3.2.3. Effects on Protein Solubility, Collagen Content, Myofibrillar Fragmentation Index (MFI), and Shear Force

**Figure 4:**
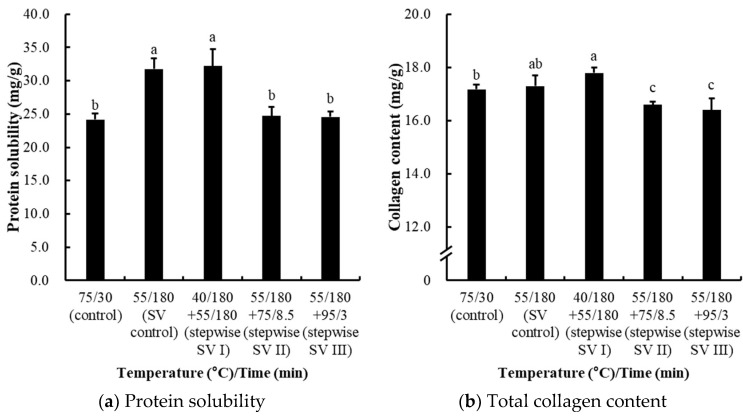

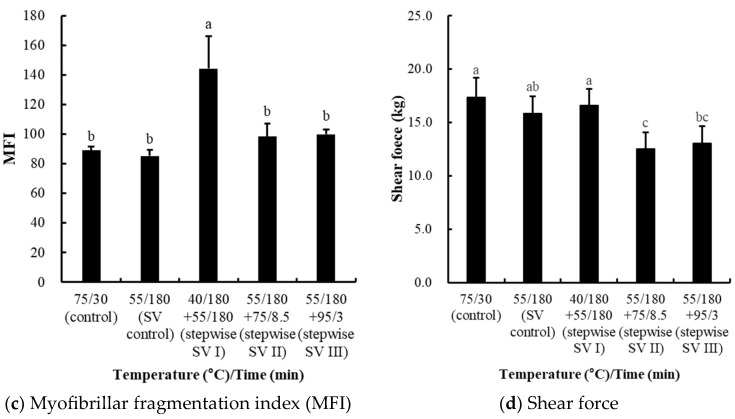
Protein solubility (**a**), collagen content (**b**), myofibrillar fragmentation index (MFI, (**c**)), and shear force (**d**) of stepwise sous-vide-cooked chicken breasts. The control was cooked at 75 °C for 30 min, and the sous-vide (SV) control was cooked at 55 °C for 3 h. The stepwise sous-vide treatments were cooked at 55 °C for 3 h before/after the combined cooking treatments at 40, 75, and 95 °C, respectively. The final core temperature of the control, stepwise SV II, and stepwise SV III was set equally to 71 °C. Error bar means standard deviation. a–c Means sharing the same letters between treatments are not significantly different (*p* ≥ 0.05).

Protein solubility is commonly used as an indicator of protein denaturation, as solubility decreases when protein aggregation occurs during the heating of meat [20,21]. In this study, the total protein solubility of chicken breast was highest in the sous-vide control and the stepwise SV I treatment, while additional heating following sous-vide cooking significantly decreased the protein solubility (*p* < 0.05; Figure 4a). Protein denaturation typically occurs in two distinct stages, depending on the temperature. Between 40 °C and 60 °C, most sarcoplasmic proteins denature [20], while at higher temperatures from 70 °C to 90 °C, the myofibrillar proteins denature, resulting in a marked decrease in protein solubility [22]. Specifically, the significant decreases in protein solubility observed in the conventional control and stepwise SV II and II treatments can be attributed to the denaturation of myofibrillar proteins at such higher temperatures. Myosin, a major myofibrillar protein, undergoes extensive denaturation at 53 °C within 3 h [23]. Similarly, actin, another critical protein, denatures by approximately 90% within 3 h at 65 °C and within just 10 min at 72 °C [24]. Thus, the lack of significant differences in protein solubility between the control and stepwise sous-vide heating treatments can be explained by the fact that myosin is almost fully denatured after heating at 55 °C for 3 h, while actin is predominantly denatured during subsequent high-temperature treatments.

The total collagen content was significantly lower in the stepwise SV II and III treatments compared to the conventional control, as well as the sous-vide control and stepwise SV I treatment (*p* < 0.05; Figure 4b). Collagen denaturation is known to begin between 53 °C and 63 °C, while solubilization into gelatin typically occurs at temperatures between 60 °C and 70 °C [19,25]. Thus, the reduced collagen content observed in the stepwise SV II and III treatments could be attributed to the higher end temperatures facilitating collagen solubilization. In fact, this current study shows that the cooking conditions of the sous-vide control might be insufficient to induce the critical denaturation and solubilization of collagen molecules, as there was no significant difference between the conventional control and the sous-vide control. Moreover, the additional heating after sous-vide cooking could contribute to collagen release through solubilization, although there was no significant difference between the different heating rates.

The myofibrillar fragmentation index (MFI) of chicken breasts subjected to stepwise sous-vide cooking was determined to evaluate the destruction intensity of myofibrillar proteins (Figure 4c). The noticeably highest MFI was observed for the stepwise SV I treatment (*p* < 0.05), which is designed to accelerate the activity of endogenous proteases in skeletal muscle. However, no significant differences were observed among the other controls and treatments, indicating that this specific temperature–time combination may have no effect on the proteolysis of muscle proteins. Previously, Wang et al. [26] reported that actomyosin dissociation in duck breast significantly increased at 50 °C, peaked at 60 °C, and then rapidly decreased at 80 °C. Similarly, Okitani et al. [27] found that the dissociation of actomyosin was highest during the first hour of heating at 60 °C, but the dissociated actin became insolubilized when the temperature was raised to 65 °C. In this study, our findings showed that the elevated MFI observed in the stepwise SV I treatment likely resulted from accelerated proteolysis and/or extended actomyosin dissociation occurring at these temperatures.

The shear force of the chicken breasts subjected to stepwise sous-vide cooking is shown in Figure 4d. The results show that all temperature-time combinations for sous-vide cooking could be effective in reducing the instrumental tenderness of cooked chicken breasts, while there was only a numerical difference between the sous-vide control/stepwise SV I treatment and the conventional control (*p* > 0.05). For the stepwise SV II and III treatments, marked decreases in shear force were found as compared to conventional control and sous-vide control (*p* < 0.05). Thus, these results indicate that the additional heating after sous-vide cooking could contribute to tender chicken breast than single-step sous-vide cooking. During the heating of meat, several transformations occur, including protein denaturation and fiber shrinkage, both of which typically contribute to an increase in shear force [21]. Generally, both the heating temperature and time affect meat tenderness, although temperature tends to have a more pronounced effect than time [19]. Barbanti and Pasquini [28] reported that improvements in tenderness are largely due to the solubilization of connective tissue, while the denaturation of myofibrillar proteins can increase the shear force. Collagen breakdown is a key factor in the tenderization of meat, and temperature plays a critical role in collagen denaturation and solubilization [29]. Thus, it is likely that collagen was effectively solubilized at these higher-end temperatures, as proven in the collagen content (Figure 4b), leading to a reduction in shear force.

#### 3.2.4. Effects on Oxidation Stability

**Figure 5:**
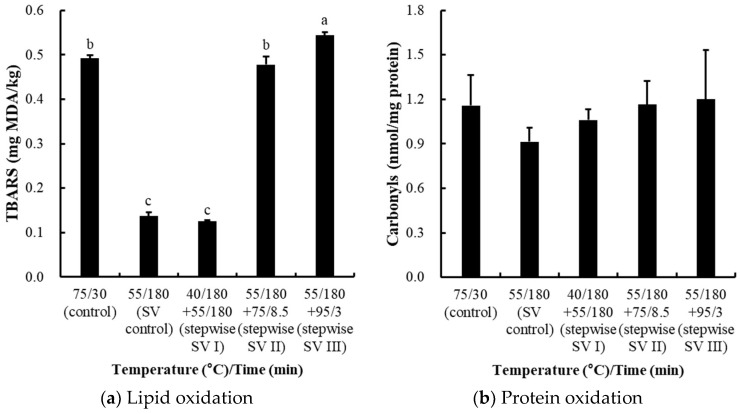
Lipid oxidation (**a**) and protein oxidation (**b**) of stepwise sous-vide-cooked chicken breasts. The control was cooked at 75 °C for 30 min, and the sous-vide (SV) control was cooked at 55 °C for 3 h. The stepwise sous-vide treatments were cooked at 55 °C for 3 h before/after the combined cooking treatments at 40, 75, and 95 °C, respectively. The final core temperature of the control, stepwise SV II, and stepwise SV III was set equally to 71 °C. Error bar means standard deviation. a–c Means sharing the same letters between treatments are not significantly different (*p* ≥ 0.05).

The TBARS values, which are indicators of lipid oxidation, were significantly lower in sous-vide control and stepwise I treatment, but the highest value was observed for stepwise SV III treatment (*p* < 0.05; Figure 5a). In terms of protein oxidation, the pattern of carbonyl formation in the samples was similar to the results for lipid oxidation, but no significant differences were found between treatments (Figure 5b). Previously, Mei et al. [30] reported that heating meat at temperatures above 60 °C to 70 °C can deactivate antioxidant enzymes, leading to increased lipid oxidation. Moreover, Xiong et al. [31] reported that enzymes such as catalase, superoxide dismutase, and glutathione peroxidase play key roles in mitigating lipid oxidation, but their activity decreases rapidly when the heating temperature exceeds 80 °C. Thus, the increase in TBARS values observed in the stepwise SV III treatment may be attributed to the inactivation of antioxidant enzymes caused by high-temperature exposure.

## 4. Conclusions

This study demonstrated that optimized sous-vide cooking at 55 °C for 3 h, as determined through response surface methodology, effectively reduced cooking loss and shear force, thereby improving the water-holding capacity and tenderness of chicken breast. Furthermore, stepwise sous-vide cooking, particularly the treatment involving additional heating at 75 °C for 8.5 min following sous-vide cooking at 55 °C (SV II treatment), significantly enhanced the tenderness without compromising moisture retention. This improvement is likely attributed to increased collagen solubilization during the second heating phase. Among the evaluated methods, SV I (40 °C + 55 °C) demonstrated benefits in preserving protein solubility and oxidative stability, while SV III (55 °C + 95 °C) resulted in greater cooking loss and lipid oxidation, suggesting that excessive heat may negatively affect meat quality. These findings suggest that a carefully controlled stepwise sous-vide approach can be an effective strategy to enhance the quality of lean poultry meat, such as chicken breast. Furthermore, these results are based on controlled laboratory conditions and may vary depending on the poultry source, packaging material, or processing environment. To facilitate industrial application, thus, further research is warranted to assess the effects of this method on the sensory properties, shelf stability, and microbiological safety of the final product.

## Figures and Tables

**Table 1:** Central faced composite design with independent (temperature and time) and response variables for optimizing sous-vide cooking conditions of chicken breasts.

Run	Coded Levels		Factor Levels		Response Variables	
	*X*_1_	*X*_2_	*X*_1_	*X*_2_	Cooking Loss (%)	Shear Force (N)
1	−1	−1	50	3.0	11.13	14.12
2	0	0	60	4.5	13.64	13.42
3	0	0	60	4.5	13.56	13.61
4	−1	0	50	4.5	11.88	14.94
5	0	0	60	4.5	13.82	13.11
6	0	1	60	6.0	14.07	13.03
7	0	0	60	4.5	11.25	13.66
8	1	0	70	4.5	14.78	12.93
9	1	1	70	6.0	15.48	11.81
10	1	−1	70	3.0	14.43	12.78
11	0	−1	60	3.0	11.39	12.94
12	−1	1	50	6.0	11.72	13.93
13	0	0	60	4.5	11.96	12.55

*X*_1_, cooking temperature (°C); *X*_2_, cooking time (h).

**Table 2:** Analysis of variance of regression coefficient calculated for optimizing sous-vide cooking conditions (temperature and time) of chicken breasts.

Source	Cooking Loss (%)			Shear Force (N)		
	Sum of Squares	Mean Square	*p* Value	Sum of Squares	Mean Square	*p* Value
Model	20.2900	4.0580	0.043	6.2516	1.2503	0.010
Linear	19.6026	9.8013	0.008	5.1776	2.5888	0.003
X_1_	16.4783	16.4783	0.004	4.9868	4.9868	0.001
X_2_	3.1243	3.1243	0.114	0.1908	0.1908	0.322
Quadratic	0.6348	0.3174	0.728	0.9218	0.4609	0.132
X_1_^2^	0.6240	0.6240	0.446	0.5070	0.5070	0.126
X_2_^2^	0.0419	0.0419	0.840	0.7513	0.7513	0.072
Interaction	0.0525	0.0525	0.821	0.1521	0.1521	0.373
X_1_X_2_	0.0525	0.0525	0.821	0.1521	0.1521	0.373
Error	6.6982	0.9569		1.1756	0.1679	
Lack of fit	1.2675	0.4225	0.818	0.3414	0.1138	0.677
Pure error	5.4307	1.3577		0.8342	0.2086	
Total	26.9881			7.4271		
Model summary						
R-squared	0.7518			0.8417		
RMSE	0.9782			0.4098		

*X*_1_, cooking temperature (°C); *X*_2_, cooking time (h); RMSE, root mean square error.

**Table 3:** pH value and color characteristics of stepwise sous-vide cooked chicken breasts.

Traits	Temperature (°C)/Time (min)					
	Conventional Control (75/30)	Sous-Vide Control (55/180)	Stepwise SV I (40/180 + 55/180)	Stepwise SV II (55/180 + 75/8.5)	Stepwise SV III (55/180 + 95/3)	Significance of *p* Value
pH value	6.23 ± 0.02	6.34 ± 0.09	6.26 ± 0.13	6.29 ± 0.09	6.32 ± 0.08	NS ^(1)^
CIE L* (lightness)	83.69 ± 0.65 ^a^	80.34 ± 1.28 ^c^	80.56 ± 0.96 ^c^	82.54 ± 0.55 ^b^	82.62 ± 0.85 ^b^	0.042
CIE a* (redness)	3.15 ± 0.46 ^b^	4.48 ± 0.38 ^a^	4.39 ± 0.44 ^a^	3.44 ± 0.51 ^b^	3.56 ± 0.54 ^b^	0.023
CIE b* (yellowness)	13.30 ± 0.33 ^ab^	13.67 ± 1.08 ^b^	13.52 ± 1.52 ^b^	13.95 ± 0.94 ^a^	13.78 ± 1.51 ^a^	0.021
Hue (°)	76.31 ± 1.68	72.25 ± 0.59	72.25 ± 3.23	75.07 ± 1.24	74.04 ± 3.36	NS
Chroma	13.35 ± 0.41	14.33 ± 1.13	14.20 ± 1.37	14.44 ± 1.03	14.33 ± 1.37	NS

The control was cooked at 75 °C for 30 min, and the sous-vide (SV) control was cooked at 55 °C for 3 h. The stepwise sous-vide treatments were cooked at 55 °C for 3 h before/after the combined cooking treatments at 40, 75, and 95 °C, respectively. The final core temperature of the control, stepwise SV II, and stepwise SV III was set equally to 71 °C. ^(1)^ NS: non-significance (*p* ≥ 0.05). ^a,b,c^ Means sharing the same letters within a row are not significantly different (*p* ≥ 0.05).

## Data Availability

The original contributions presented in the study are included in the article; further inquiries can be directed to the corresponding author.
